# The histone methyltransferase G9a regulates tolerance to oxidative stress–induced energy consumption

**DOI:** 10.1371/journal.pbio.2006146

**Published:** 2019-03-12

**Authors:** Human Riahi, Carlijn Brekelmans, Sarah Foriel, Sarah H. Merkling, Taylor A. Lyons, Pavel M. Itskov, Tjitske Kleefstra, Carlos Ribeiro, Ronald P. van Rij, Jamie M. Kramer, Annette Schenck

**Affiliations:** 1 Department of Human Genetics, Donders Institute for Brain, Cognition and Behaviour, Radboud University Medical Center, Nijmegen, the Netherlands; 2 Khondrion BV, Nijmegen, the Netherlands; 3 Radboud University Medical Center, Nijmegen, the Netherlands; 4 Department of Medical Microbiology, Radboud Institute for Molecular Life Sciences, Radboud University Medical Center, Nijmegen, the Netherlands; 5 Department of Physiology and Pharmacology, Schulich School of Medicine and Dentistry, Western University, London, Ontario, Canada; 6 Department of Biology, Faculty of Science, Western University, London, Ontario, Canada; 7 Division of Genetics and Development, Children’s Health Research Institute, London, Ontario, Canada; 8 Champalimaud Centre for the Unknown, Lisbon, Portugal; 9 Sechenov First Moscow State Medical University, Moscow, Russia; Duke University, United States of America

## Abstract

Stress responses are crucial processes that require activation of genetic programs that protect from the stressor. Stress responses are also energy consuming and can thus be deleterious to the organism. The mechanisms coordinating energy consumption during stress response in multicellular organisms are not well understood. Here, we show that loss of the epigenetic regulator G9a in *Drosophila* causes a shift in the transcriptional and metabolic responses to oxidative stress (OS) that leads to decreased survival time upon feeding the xenobiotic paraquat. During OS exposure, G9a mutants show overactivation of stress response genes, rapid depletion of glycogen, and inability to access lipid energy stores. The OS survival deficiency of G9a mutants can be rescued by a high-sugar diet. Control flies also show improved OS survival when fed a high-sugar diet, suggesting that energy availability is generally a limiting factor for OS tolerance. Directly limiting access to glycogen stores by knocking down glycogen phosphorylase recapitulates the OS-induced survival defects of G9a mutants. We propose that G9a mutants are sensitive to stress because they experience a net reduction in available energy due to (1) rapid glycogen use, (2) an inability to access lipid energy stores, and (3) an overinduced transcriptional response to stress that further exacerbates energy demands. This suggests that G9a acts as a critical regulatory hub between the transcriptional and metabolic responses to OS. Our findings, together with recent studies that established a role for G9a in hypoxia resistance in cancer cell lines, suggest that G9a is of wide importance in controlling the cellular and organismal response to multiple types of stress.

## Author summary

Stress responses require proper activation of genetic programs to protect the organism from the stressor. However, the mechanisms controlling energy consumption during stress responses are not well understood. Here, we investigate the role of epigenetic modifier G9a in regulating metabolism and gene transcription during oxidative stress responses in *Drosophila*. Flies lacking G9a show a shift in the metabolic and transcriptional responses to oxidative stress, leading to decreased stress tolerance despite intact oxidative stress defense mechanisms. During oxidative stress exposure, G9a mutants show overactivation of stress response and many other genes, rapid depletion of glycogen energy stores, and an inability to access lipid energy stores. The increased susceptibility of G9a mutant flies to oxidative stress can be rescued simply by providing extra sugar. This suggests that G9a mutants are sensitive to stress because of reduced access to immediately available energy. Wild-type flies also become more tolerant to oxidative stress when they are fed extra sugar, whereas blocking energy access by genetically reducing a key metabolic enzyme leads to oxidative stress sensitivity. Though the genetic response to oxidative stress has long been appreciated, our study emphasizes the importance of energy metabolism for stress tolerance and identifies the histone methyltransferase G9a as an important player regulating both.

## Introduction

The ability of an organism to sense and adapt to changes in the environment is essential for survival. In particular, harmful environmental challenges require acute responses to avoid cellular and organismal damage [1]. The coordinated regulation of stress response genes coupled with a reallocation of cellular energy use is required to implement effective cellular stress responses [2,3]. In extreme cases, translation of non-stress-related proteins is repressed, and cellular energy stores become primarily dedicated to the stress response, at the expense of other normal cellular processes [4]. Little is known about the regulators that safeguard an appropriate amplitude of stress response and ensure sufficient cellular resources to execute an effective defense.

Conserved defense mechanisms have evolved to counteract stress such as heat shock, DNA damage, and oxidative stress (OS) [1,5]. Exposure to xenobiotics such as paraquat or hydrogen peroxide (H_2_O_2_) can lead to increased accumulation of reactive oxygen species (ROS). Increased ROS triggers multiple signaling pathways, which activate key transcription factors such as c-Jun N-terminal kinase (JNK) [6], activator protein 1 (AP-1) (D-Fos/D-jun) [7], forkhead box O (FoxO) [8], and activating transcription factor 3 (Atf-3) [9]. These transcription factors induce the expression of ROS scavengers (superoxide dismutases [SODs], catalases, glutathione peroxidases, glutathione S-transferases) and genes involved in the repair of ROS-mediated damage (peroxiredoxins, proteasomal components, DNA repair machinery) [10,11].

Recent studies have indicated that chromatin regulators are critical factors in mediating the cellular response to stress [12]. Chromatin modifiers of the evolutionarily conserved G9a/euchromatin histone methyltransferase (EHMT) protein family mediate histone H3 lysine 9 dimethylation (H3K9me2) within euchromatic regions the genome [13]. Recently, it has been shown that G9a is required for hypoxia resistance during the rapid proliferation of cancer cells in culture [14,15]. At the organismal level, G9a is important in mediating responses to various environmental insults and stimuli, including viral infection [16], starvation [17,18], cocaine [19,20], and learning [21,22]. Previously, we characterized putative genomic H3K9me2 target sites of G9a in *Drosophila* larvae. These G9a target sites were enriched at genes that are regulated in environmentally induced processes requiring immediate responses, including memory, immune response, and response to OS [22]. Whereas the former were demonstrated to be predictive for defects in learning and memory [22] and immune responses to virus infection [16] of G9a-null mutant flies, the biological relevance of *Drosophila* G9a OS-related targets has remained elusive.

In the present study, we demonstrate an essential role for G9a in OS tolerance. Our data suggest that G9a mutants experience an overactivated stress response, elevated glycogen use, and an inability to access lipid energy reserves, resulting in premature death due to reduced net energy availability. This defines G9a as an important regulator of transcriptional and metabolic homeostasis that is required for an optimal metabolic response to stress.

## Results

### G9a is required for optimal survival in response to paraquat-induced OS

**Fig 1:**
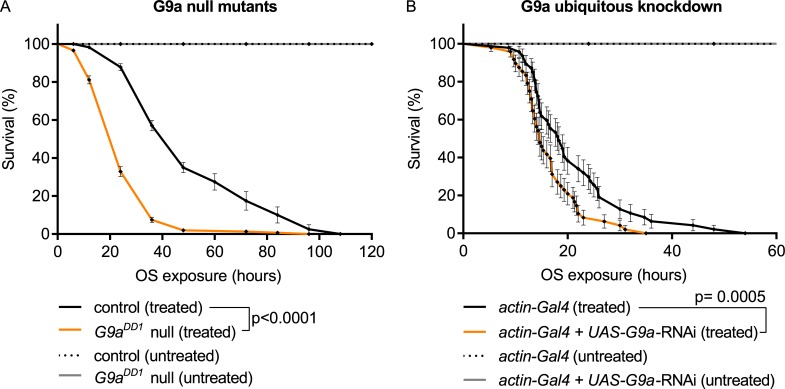
G9a is required for resistance to paraquat-induced OS. (A) Survival curves of G9a-null mutants and controls upon paraquat-induced OS exposure (treated) show reduced survival in *G9a*^*DD1*^ mutants (median survival time: *G9a*^*DD1*^ mutants, treated: 24 h, *n* = 324; versus control, treated, 48 h, *n* = 330; *p* < 0.0001). G9a mutants and controls show normal longevity without OS exposure (untreated) during the time course of the experiment (*G9a*^*DD1*^, untreated *n* = 632; control, untreated *n* = 697). (B) Survival curves of G9a ubiquitous knockdown flies show reduced survival upon OS exposure (*actin-Gal4* + *UAS-G9a*-RNAi, treated) compared to the controls (*actin-Gal4*, treated) (median survival time: *actin-Gal4* + *UAS-G9a*-RNAi, treated, 14 h, *n* = 48 versus *actin-Gal4*, treated, 18 h, *n* = 48; *p* = 0.007) and normal longevity when untreated. The ubiquitously expressed actin driver was combined with a *UAS-G9a* RNAi construct (*actin-Gal4* + *UAS-G9a-RNAi*) to knock down G9a expression or crossed to the isogenic background of the RNAi construct to generate the isogenic control (*actin-Gal4*). Survival curves showing percent survival over time and SE were plotted using Graphpad, and *p*-values were obtained using the Gehan-Breslow-Wilcoxon statistical test. All experiments were independently replicated at least three times. The numerical data depicted in this figure can be found in S5 Data. OS, oxidative stress; RNAi, RNA interference; SE, standard error.

To investigate whether G9a is required for OS response, we exposed G9a-null mutants (*G9a*^*DD1*^ and *G9a*^*DD2*^) and an isogenic control strain [22] to paraquat, a potent inducer of OS [23]. Scoring survival of the three genotypes over time, we found that G9a mutants showed reduced survival upon paraquat exposure, dying dramatically faster than the controls. In contrast, untreated G9a-null mutants and controls showed full viability over the time course of the experiment (Fig 1A and S1A Fig). To independently verify this finding, we also generated G9a knockdown flies and exposed them to paraquat. Knocking down G9a using the ubiquitous *actin-Gal4* driver and a previously validated *G9a* RNA interference (RNAi) line [24] also led to a significant reduction in median survival time during exposure to paraquat compared to controls. Untreated G9a-knockdown and control flies were viable over the time course of the experiment (Fig 1B). The observation that two *G9a*-null alleles and G9a-knockdown flies show reduced survival in response to paraquat suggests that G9a may play a role in OS response. G9a mutant flies were also sensitive to the OS-inducing agent menadione sodium bisulfide (MSB) (S1B Fig), demonstrating that the sensitivity to OS is not specific to paraquat.

### G9a mutants show a highly augmented transcriptional response to OS

**Fig 2:**
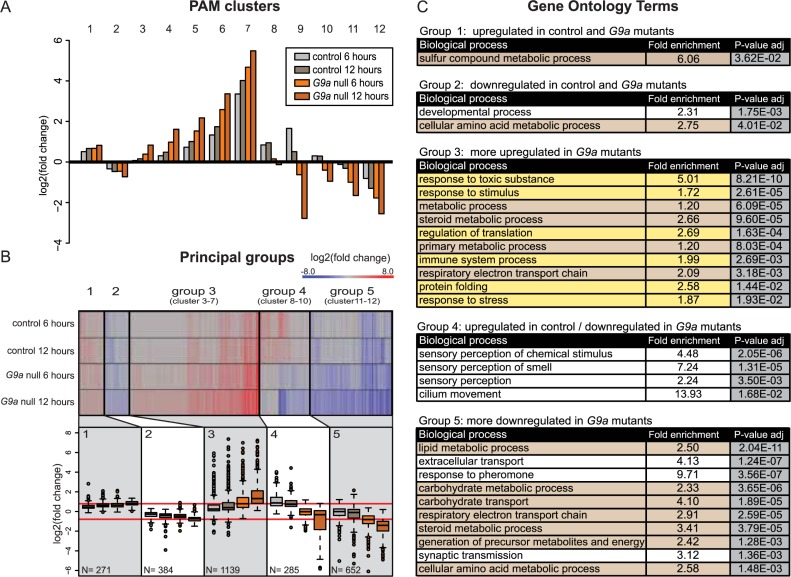
G9a mutants show highly augmented transcriptional response of genes regulating stress defenses and metabolism. (A) PAM clustering of differentially expressed genes base on log2 fold change values obtained from differential expression analysis in four pairwise comparisons. (B) Heatmap and boxplots of log2 fold changes of differentially expressed genes combined into five principle groups derived from clusters with similar patterns of differential expression. The five principle groups show up-regulation in G9a mutants and controls (group 1, cluster 1), down-regulation in G9a mutants and controls (group 2, cluster 2), more up-regulation in G9a mutants compared to controls (group 3, clusters 3–7), up-regulated in controls and down-regulated in G9a mutants (group 4, clusters 8–10), and more down-regulation in G9a mutants than in controls (group 5, clusters 11 and 12). The number of genes in each group is indicated. (C) Gene ontology analysis showing the top enriched biological processes sorted by adjusted (Bonferroni-corrected) *p*-value in each of the five principal groups, indicating enrichment in stress response genes (highlighted in yellow) and metabolic genes (highlighted in brown). The numerical data depicted in this figure can be found in S5 Data. PAM, partitioning around medoids.

Previous chromatin immunoprecipitation sequencing (ChIP-seq) profiling of H3K9me2, the epigenetic mark deposited by G9a, in G9a mutants versus control larvae revealed that genes implicated in OS are enriched among putative G9a target genes. Based on this finding and the increased susceptibility of G9a mutants to OS, we hypothesized that G9a may be required for an appropriate transcriptional response to OS. To address this hypothesis and to uncover specific mechanisms underlying OS sensitivity of G9a mutant flies, we assessed gene expression changes in G9a mutants over a time course of OS exposure. For this, we generated transcriptome profiles by RNA sequencing (RNA-seq) of G9a mutant and control heads at 0, 6, and 12 h after paraquat exposure. We mapped reads to the *Drosophila* reference genome and created normalized count data (see Materials and methods and S1 Data). Euclidean sample-to-sample distance (S2A Fig) showed that (1) biological duplicates cluster together, (2) controls and G9a mutants with no OS exposure cluster apart from each other, (3) samples with OS exposure cluster apart from samples with no OS exposure, and (4) G9a mutant samples after 6 and 12 h OS exposure cluster apart from the control samples with OS exposure. These findings suggest that there are differences in the global transcriptional response to OS in G9a mutants compared to controls. Principal component analysis confirms that sample duplicates cluster closely together. It also illustrates that transcriptional changes upon OS are more dramatic in the mutants than in the controls (S2B Fig). To identify differentially expressed (DE) genes, we used DESeq [25], with cutoffs of ≥1.5-fold change and *p*-adj ≤ 0.05. We performed four pairwise comparisons: 0 h versus 6 h OS exposure and 0 h versus 12 h OS exposure in controls and in G9a mutants. We found 2,731 genes to be differentially expressed in at least one of the four pairwise comparisons (S2A Data). To reveal patterns in global gene expression changes among the two genotypes during OS exposure, we used the partitioning around medoids (PAM) R package [26] to identify 12 clusters of genes with similar expression changes in response to OS (Fig 2A). Some of these 12 clusters showed a similar pattern across the four conditions, varying mostly by the amplitude of the changes. These were pooled, resulting in five principal groups (Fig 2B and S2A Data). Genes in group 1 (271 genes, cluster 1) were up-regulated upon OS exposure in both G9a mutants and controls to a similar extent. Group 2 (384 genes, cluster 2) represents genes that were down-regulated in both G9a mutants and controls. Group 3 (1,139 genes, clusters 3–7), the largest group, includes genes that were induced by OS in control and mutant conditions but to a larger extent in G9a mutants. Group 4 (285 genes, clusters 8–10) represents genes that were up-regulated in the controls but down-regulated in G9a mutants. Group 5 (652 genes, clusters 11 and 12) contains genes that were down-regulated in the control and mutant conditions but to a larger extent in G9a mutants.

Next, we performed gene ontology (GO) enrichment analysis to obtain a global understanding of the biological function associated with the genes in the five principle groups described in Fig 2B (complete GO statistics are shown in S3A Data). We identified several GO terms related to stress response in the large group 3 (Fig 2C, highlighted in yellow, e.g., response to toxic substances, response to stimulus, regulation of translation, immune system process, protein folding, response to stress), which contained genes that were up-regulated in response to OS but more up-regulated in G9a mutants (Fig 2A and 2B). Thus, G9a mutants show an augmented transcriptional response to OS. In addition, many metabolic terms were enriched (Fig 2C, highlighted in brown). This was especially evident for group 5 genes (Fig 2C, e.g., lipid metabolic processes, carbohydrate metabolic processes, carbohydrate transport, steroid metabolic processes, generation of precursor metabolites, and energy and cellular amino acid metabolic processes), which were down-regulated in response to stress but more down-regulated in G9a mutants (Fig 2A and 2B).

We also investigated an alternative RNA-seq data analysis approach by performing pairwise comparisons of transcriptional changes in G9a mutants versus control at each of the three time points: 0, 6, and 12 h of OS exposure (cutoffs of ≥1.5-fold change and *p*-adj ≤ 0.05). Using this approach, we identified 2,600 DE genes (S2B Data). By PAM clustering of DE genes, as described above, we again identified five principle gene expression groups (S3 Fig). We found similar patterns of expression changes and comparable representation of biological process within five different principal groups (S3 Fig and S3B Data). For example, group 3 of this alternative analysis contained many stress genes that were up-regulated in G9a mutants only at 6 and 12 h after OS but not at 0 h in steady-state conditions. Group 5 of the alternative analysis contained many metabolic genes that were down-regulated in G9a mutants after 6 and 12 h of OS exposure but not at 0 h in steady-state conditions (S3 Fig). Taken together, we consistently see augmented activation of stress response genes and reduced expression of metabolic genes in G9a mutants after exposure to OS.

### Transcriptional overactivation of OS defense mechanisms in G9a mutants

**Fig 3:**
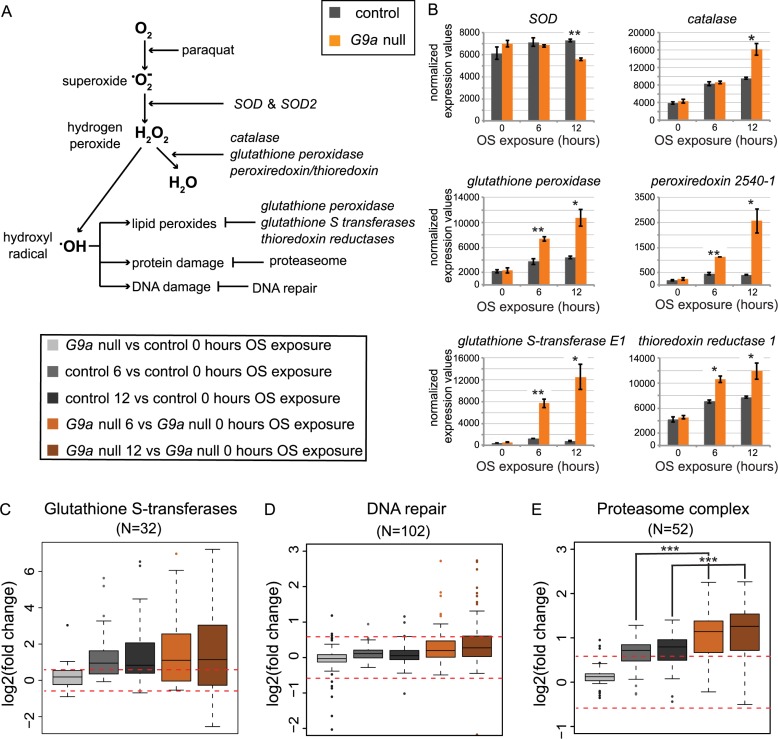
Enhanced expression of antioxidant and ROS damage pathway genes in G9a mutants upon OS exposure. (A) Schematic diagram of enzymes crucial for ROS elimination and prevention of ROS-mediated damage. (B) Normalized expression values for selected genes involved in ROS elimination and ROS-mediated damage in G9a mutants and controls at 0, 6, and 12 h after OS induction. Significance was determined using Student *t* test. (C–E) Boxplots showing log2 fold changes for selected groups of genes encoding glutathione S-transferases, peroxisomal proteins, and the DNA repair machinery. Fold changes were derived from the following pairwise comparisons: G9a mutant versus control after 0 h OS (light gray), control 0 versus 6 (dark gray) and 12 h (black) of OS, and G9a mutant 0 versus 6 (orange) and 12 h (brown) of OS. Statistical comparisons between groups were performed using a Wilcoxon signed-rank test. ***p < 0.001; **p < 0.01; *p < 0.05. The numerical data depicted in this figure can be found in S5 Data. H_2_O_2_, hydrogen peroxide; OS, oxidative stress; ROS, reactive oxygen species; *SOD*, *superoxide dismutase*.

We initially hypothesized that the strongly reduced survival of G9a mutants in response to OS may be due to the inability to initiate the transcriptional defense mechanisms protecting against OS. It was thus surprising to identify stress response genes to be enriched in group 3, characterized by an exaggerated transcriptional response in G9a mutants (Fig 2). We therefore further analyzed specific genes encoding proteins that function in neutralizing ROS and oxidative damage (Fig 3A). SOD catalyzes transformation of oxygen radicals (^•^O^−^_2_) into H_2_O_2_. Catalase, glutathione peroxidases, peroxiredoxins, and thioredoxins help to reduce H_2_O_2_ to water. Examining OS-induced expression changes of these enzymes, we found similar expression in G9a mutants and controls at steady state. Upon OS exposure, we observed an increase in mRNA levels after 6 h and even more after 12 h of OS exposure, a response that was augmented in G9a mutants (Fig 3B). The most striking examples for augmented induction by OS are *glutathione S-transferase E1* (*GstE1*) (fold increase at 12 h after OS = 1.8 in controls versus 22.1 in G9a mutants, *p* = 0.017, Fig 3B, bottom left) and *peroxiredoxin 2540–1* (*Prx2450-1*) (fold increase at 12 h after OS = 2.2 in controls versus 10.5 in G9a mutants, *p* = 0.025, Fig 3B, middle right). These expression differences were validated in independent quantitative real-time PCR (RT-qPCR) experiments (S4A Fig). *GstE1*, *Prx2450-1*, and *Catalase* (*Cat*) were previously predicted to be G9a targets by H3K9me2 ChIP-seq [22], suggesting that G9a might be required to buffer the stress-induced induction of some OS response genes. These data show that G9a mutants are not defective in their ability to induce expression of OS response genes but rather show increased induction.

Cellular ROS can react with and cause damage of lipids, DNA, and proteins. We therefore further surveyed the expression of protein groups that counteract ROS-mediated damage. These included glutathione S-transferases, which are responsible for detoxifying peroxidized lipids (Fig 3C), DNA damage repair machinery (Fig 3D), and genes encoding components of the proteasome complex, a key organelle in clearing damaged proteins (Fig 3E). In general, genes involved in these processes are not differentially expressed in G9a mutants under steady-state conditions (Fig 3C–3E, G9a null versus control after 0 h OS). At 6 h and 12 h OS, glutathione S-transferase genes show a trend toward increased expression in controls, which is somewhat augmented in G9a mutants (Fig 3C). DNA repair genes show no induction after OS in controls or G9a mutants, with the exception of some outliers (Fig 3D). For genes encoding components of the proteasome, we observed a trend toward increased expression in controls after OS exposure, and this increase was significantly higher in G9a mutants (Fig 3E, G9a null versus controls after 6 h OS: *p* = 0.0012, G9a null versus control after 12 h OS: *p* = 0.0011). Together, our data indicate that G9a mutants show an intact and even exaggerated transcriptional response of genes implicated in OS defense.

### No accumulation of ROS or ROS-induced damage in G9a mutants

**Fig 4:**
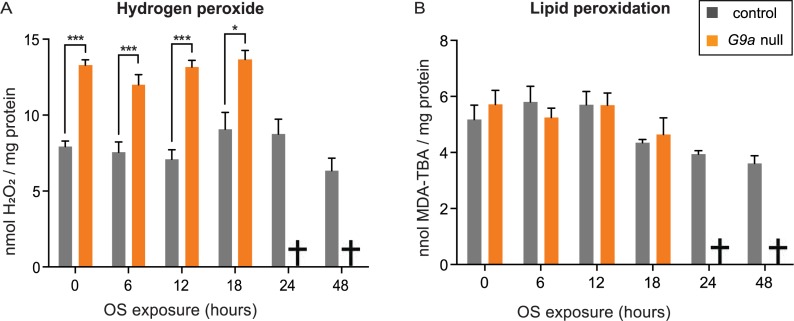
No accumulation of ROS or ROS-mediated damage during OS exposure in G9a mutants and controls. (A) Hydrogen peroxide and (B) lipid peroxidation levels in G9a mutants and controls over a time course of OS exposure. Measurements could not be obtained for the G9a mutants at 24 and 48 h time points, as most flies were already dead (indicated by cross). Bar graphs showing mean values and SEM were generated using Graphpad, and *p*-values were obtained using multiple *t* tests with FDR correction. ***p < 0.001; *p < 0.05. The numerical data depicted in this figure can be found in S5 Data. FDR, false discovery rate; OS, oxidative stress; ROS, reactive oxygen species; MDA, malondialdehyde.

Having identified that the transcription of OS defense genes was enhanced in G9a mutants, we set out to measure markers of OS and oxidative damage in G9a mutants and controls upon OS induction. We measured H_2_O_2_ levels, an intermediate ROS metabolite used as a marker for ROS levels. G9a mutants already had significantly higher levels of H_2_O_2_ at steady state (0 h OS) compared to controls (Fig 4A). However, H_2_O_2_ remained unchanged for up to 18 h after OS exposure, which was the latest time point at which we were able to collect sufficient living G9a mutant animals to perform measurements. We also estimated lipid peroxidation as a marker for oxidative damage, by quantifying malondialdehyde (MDA) levels in G9a mutants and controls (Fig 4B). We observed similar levels of MDA in both genotypes at steady state (0 h OS) and no changes in MDA levels upon OS induction. This indicates that G9a mutants are not defective in clearing ROS or eliminating molecules that are damaged by ROS, in agreement with the transcriptional data. In addition, we tried to rescue the G9a mutant OS sensitivity by feeding the antioxidants vitamin E and glutathione at concentrations that have previously been shown to counteract harmful effects of OS [27,28]. However, we did not see any beneficial effects of these antioxidants in either G9a mutants or controls (S5 Fig), suggesting that ROS clearance is not limiting for survival under these conditions.

### G9a mutants show altered regulation of metabolic genes driving energy storage and usage

**Fig 5:**
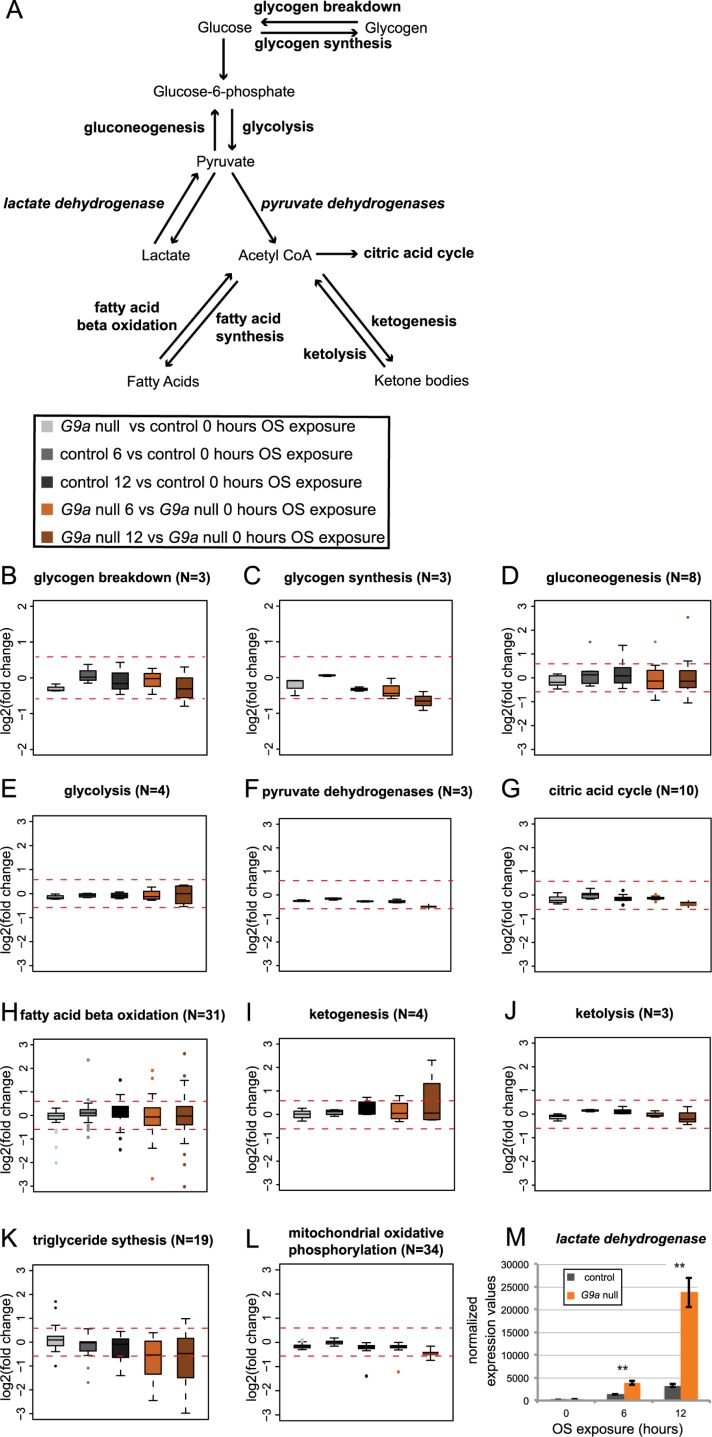
Altered expression of metabolic enzymes regulating energy storage and release in G9a mutants upon OS exposure. (A) Simplified schematic diagram of energy metabolism. (B-L) Boxplots showing log2 fold changes for selected groups of genes encoding enzymes involved in glycogen breakdown (B), glycogen synthesis (C), gluconeogenesis (D), glycolysis (E), pyruvate dehydrogenases (F), citric acid cycle (G), fatty acid beta oxidation (H), ketogenesis (I), ketolysis (J), triglyceride synthesis (K), and mitochondrial oxidative phosphorylation (L). Fold changes were derived from the following pairwise comparisons: G9a mutant versus control after 0 h OS (light gray), control 0 h versus 6 h (dark gray) and 12 h (black) of OS, and G9a mutant 0 h versus 6 h (orange) and 12 h (brown) of OS. (M) Normalized expression values for lactate dehydrogenase. *p*-Values were obtained using a Student *t* test. **p < 0.01. The numerical data depicted in this figure can be found in S5 Data. OS, oxidative stress.

The results obtained above strongly suggested mechanisms other than an impaired stress response to underlie reduced survival of G9a mutants in response to OS. We therefore turned to the second predominant biological theme of misregulated genes: genes involved in metabolism. We examined OS-induced expression changes for genes encoding the main enzymes that drive energy use and storage (Fig 5A and S6 and S7 Figs). In G9a mutants at steady state, metabolic genes showed very little misregulation (Fig 5B–5M and S7 Fig; G9a mutants versus controls after 0 h OS), with the exception of a few genes involved in the regulation of fatty acid beta oxidation and triglyceride synthesis (S6 Fig). Upon OS induction, there were no global changes in expression of genes involved in glycogen metabolism, gluconeogenesis, glycolysis, pyruvate dehydrogenases, citric acid cycle, ketogenesis, ketolysis, or mitochondrial oxidative phosphorylation (Fig 5B–5L). There were also no significant differences in expression of these genes in G9a mutants compared to controls after OS induction (S7 Fig). However, we noticed that specific genes regulating fatty acid beta oxidation showed highly diverging expression changes (in either direction, up and down) in response to OS in the G9a mutants compared to the controls (Fig 5H and S6 and S7 Figs). Genes involved in triglyceride synthesis showed a clear trend toward augmented down-regulation in G9a mutants upon both 6 h and 12 h of OS (Fig 5K and S6 and S7 Figs). The most dramatic effect was observed for the *Lactate dehydrogenase* (*Ldh*) gene, the sole enzyme responsible for the conversion of pyruvate to lactate, which was significantly up-regulated in controls upon OS induction and dramatically overinduced in G9a mutants (fold increase at 12 h after OS = 17.91 for controls, 95.83-fold for G9a mutants, *p* = 0.012, Fig 5M). Like the hyperactivated genes operating in OS defense (*GstE1*, *Prx2450-1*, and *Cat*), *Ldh* was previously identified as a potential direct target gene of G9a [22]. We validated the identified expression changes for several metabolic genes, including *Ldh*, in an independent RT-qPCR experiment (S4 Fig).

### G9a mutants show accelerated glycogen consumption and failure to mobilize triglycerides during OS exposure

**Fig 6:**
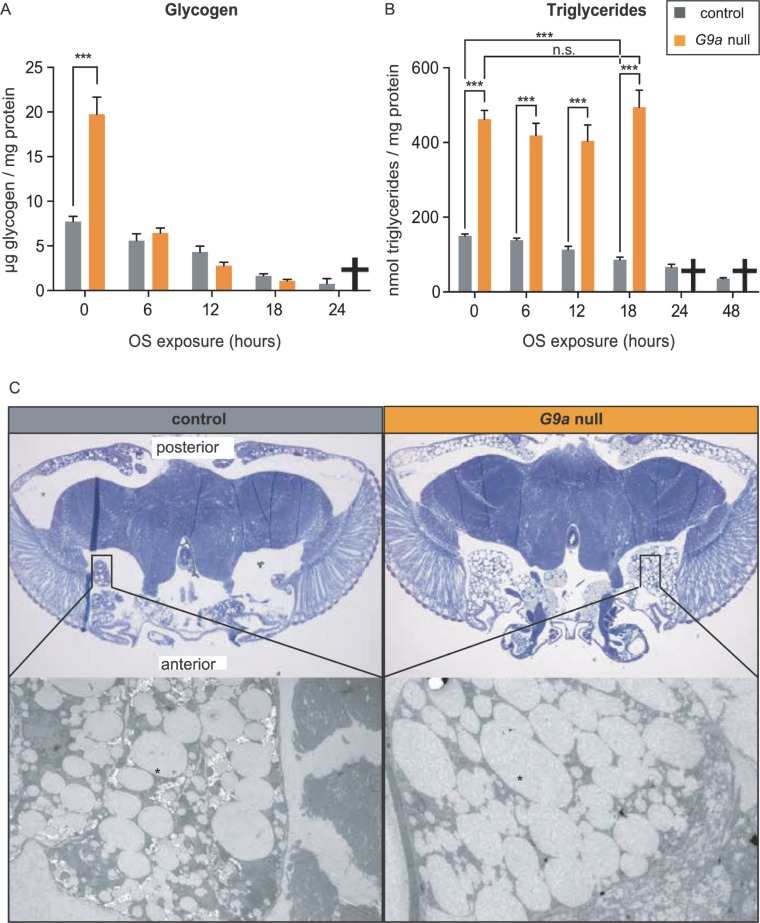
Altered energy stores and accelerated energy consumption in G9a mutants during OS exposure. (A) Glycogen and (B) triglyceride levels in G9a mutants and controls over a time course of OS exposure. Bar graphs showing mean values and SEMs were generated using Graphpad, and *p*-values were obtained using multiple *t* tests with FDR correction. ***p < 0.001; n.s., *p* > 0.05. Metabolites could not be measured for the G9a mutants at 24 and 48 h time points, as the vast majority of flies had died (indicated by a cross). (C) Images from toluidine blue–stained sections of G9a mutant and controls heads obtained using bright-field 10× magnification (top) and scanning electron (bottom) microscopy. Fat body tissues (black rectangle) lateral anterior and posterior are enlarged in G9a mutants. Electron microscopy sections show lipid droplets (indicated by star), which are larger in G9a mutants than in controls. The numerical data depicted in this figure can be found in S5 Data. FDR, false discovery rate; n.s., not significant; OS, oxidative stress.

Having observed misregulation of several genes involved in fat metabolism (see fatty acid beta oxidation and triglyceride synthesis, Fig 5, S6 and S7 Figs) and energy use (*Ldh*, Fig 5M) in G9a mutant heads at steady state and upon OS induction, we asked whether G9a mutants show altered energy use during OS exposure. In controls, glycogen stores were gradually depleted at a rate of −0.29 normalized glycogen units (ngu)/h over the time course of OS exposure (Fig 6A). Although glycogen levels were 2.5-fold higher in G9a mutants at steady state compared to controls (G9a mutants = 19.75 ngu, controls = 7.73 ngu, *p* < 0.0001, Fig 6A), glycogen stores got depleted at a much higher, exponential rate (−0.99 ngu/h) in G9a mutants, reaching values lower than in controls after 12 h of OS. These differences in glycogen metabolism might be mediated through posttranslational modifications rather than transcriptional changes, since mRNA levels of glycogen regulators were overall not significantly different in G9a mutants versus controls (Fig 5 and S7 Fig).

Similar to glycogen energy stores, triglyceride levels were also highly increased in G9a mutants at steady state when compared to controls (G9a mutants = 462.81 normalized triglyceride units [ntu], controls = 150.06 ntu, *p* < 0.0001, Fig 6B). Upon OS exposure, triglyceride levels gradually decreased in control flies at a rate of −4.68 ntu/h (controls 0 h versus 18 h, *p* < 0.001). In contrast, triglyceride levels in the G9a mutants did not decrease during OS exposure (Fig 6B). In agreement with the measured high triglyceride levels in G9a mutants, histological sectioning revealed that G9a mutant heads, in comparison to controls, presented with a striking increase in overall pericerebral fat body size and in lipid droplet diameter (Fig 6C). Thus, G9a mutant heads showed a dramatic increase in steady-state energy stores and showed altered use of these stores during the OS response. This is consistent with the observed misregulation of genes involved in lipid metabolism in G9a mutants at steady state and during OS exposure (Fig 5, S6 and S7 Figs).

We asked whether the observed differences of energy stores between G9a mutants at steady state and during OS exposure are due to differences in feeding. We found that G9a mutants have normal food intake; they eat neither more at steady state nor less upon OS exposure, as could be expected in light of increased steady-state energy stores and quickly declining glycogen (S8 Fig). Taken together, these data show that *G9a* mutants rapidly used up glycogen during the OS response, and they failed to mobilize triglycerides. The data also indicate that the observed differences in energy stores result from transcriptional and metabolic dysregulation that occurs despite normal food intake.

### G9a is required for proper OS response in tissues that regulate energy homeostasis

**Fig 7:**
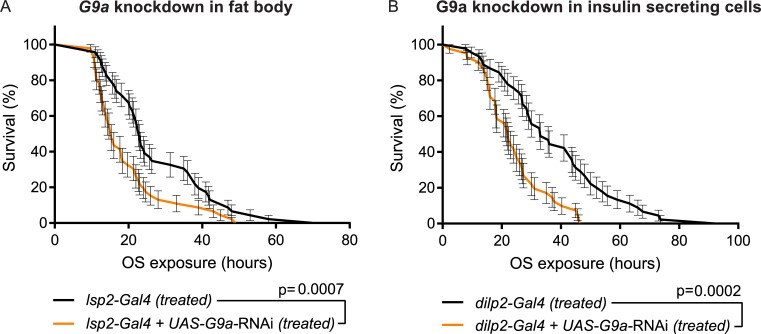
Tissue-specific requirement for G9a in OS response. Kaplan-Meier survival curves comparing G9a-knockdown flies to controls. Knockdown was targeted to (A) fat body and (B) dilp2-expressing cells. (A) Survival curves of flies with fat body–specific G9a knockdown (*lsp2-Gal4* + *UAS*-*G9a*-RNAi) and controls (*lsp2-Gal4*) (median survival time: *lsp2-Gal4* + UAS-*G9a*-RNAi, 15 h, *n* = 46 versus *lsp2-Gal4*, 23 h, *n* = 46; *p* = 0.0007). (B) Survival curves of flies with insulin-secreting cell-specific *G9a* knockdown (*dilp2-Gal4* + *UAS*-*G9a*-RNAi) and controls (*dilp2-Gal4*) (median survival time: *dilp2-Gal4* + *UAS*-*G9a*-RNAi, 22 h, *n* = 41 versus *dilp2-Gal4*, 33 h, *n* = 45; *p* = 0.0002). Fat body–or insulin-secreting cell–specific drivers were combined with a *UAS-G9a* RNAi construct (*lsp2-Gal4* + *UAS-G9a-RNAi* [A], *dilp2-Gal4* + *UAS-G9a RNAi* [B]) to repress *G9a* expression in a tissue/cell-specific manner or crossed to the isogenic background of the RNAi lines to generate the isogenic controls. Survival curves showing percent survival and SE over time were plotted using Graphpad, and *p*-values were obtained using the Gehan-Breslow-Wilcoxon test. All experiments were independently replicated at least three times. The numerical data depicted in this figure can be found in S5 Data. OS, oxidative stress; RNAi, RNA interference; SE, standard error.

Because the results above revealed abnormal energy consumption and metabolic dysregulation in G9a mutants, we investigated the role of G9a in tissues regulating energy homeostasis. We performed tissue-specific knockdown of G9a in fat body tissue using the *lsp2-Gal4* driver, in insulin-secreting cells using the *dilp2-Gal4* driver, and in all neurons using the *elav-Gal4* driver (Fig 7A and 7B and S9 Fig). The *Drosophila* fat body is the main organ for energy storage and important for energy homeostasis during growth and development as well as in immediate energy release in response to environmental stressors such as starvation, infection [16], and OS [3,29]. G9a knockdown in the fat body reduced survival upon OS, demonstrating a crucial function of G9a in fat body tissue during OS induction by paraquat (Fig 7A). *Drosophila* insulin-like peptide 2 (dilp2) neurons have a neuroendocrine function and are responsible for orchestrating hormonal regulation of energy storage and release via secretion of dilps, which act on fat body, muscles, gut, and other organs involved in uptake, storage, synthesis, and release of energy [30]. Decreasing G9a levels in dilp2-expressing neurons also resulted in reduced OS resistance (Fig 7B). In contrast, panneuronal knockdown using *elav-Gal4* did not have an effect (S9 Fig), possibly because *elav*-driven Gal4 is not as strongly expressed as *dilp2*-driven Gal4 in dilp2-expressing neurons. Taken together, the data suggest that G9a safeguards energy homeostasis during OS in tissues with a known role in mediating organismal energy homeostasis.

### Energy availability is a key determinant of OS resistance

Finally, we asked whether energy could be a limiting factor for survival in our paraquat-induced OS regime and whether abnormal energy consumption in G9a mutants underlies their premature death in response to OS. We addressed these questions by manipulation of glucose availability in G9a mutant and control animals.

**Fig 8:**
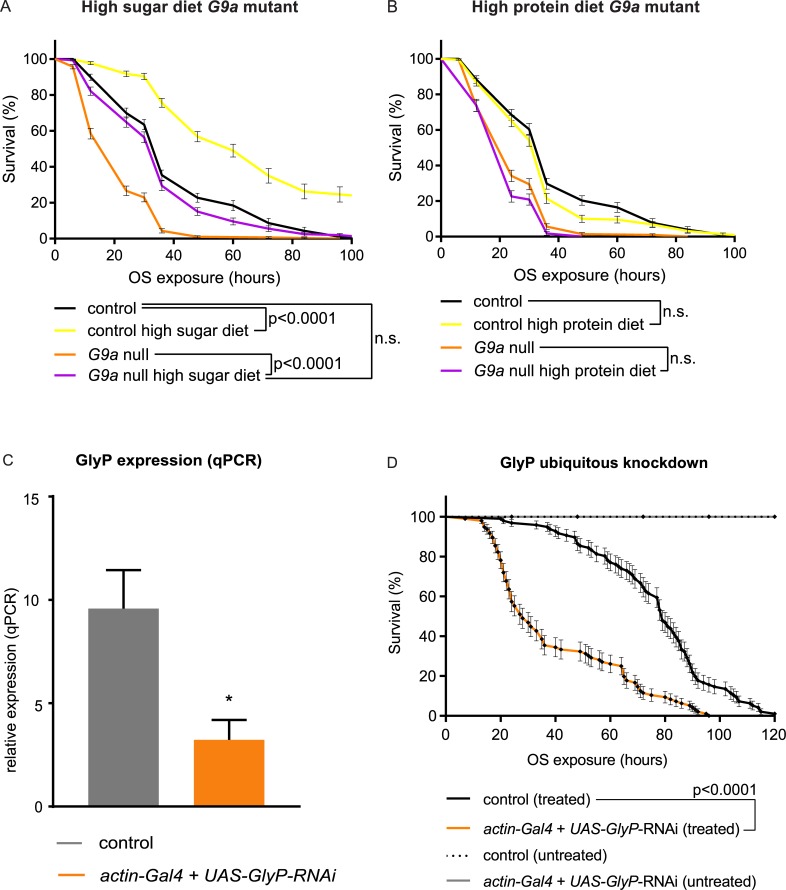
OS-mediated survival defects of G9a mutants can be rescued by a high-sugar diet and are recapitulated by inhibition of glycogen breakdown. (A, B) Survival curves of G9a mutants and controls fed or not with a high-sugar (A) or high-protein (B) diet during OS exposure. (C) *GlyP* relative expression in ubiquitous GlyP-knockdown flies (*actin-Gal4* + *UAS*-*GlyP*-RNAi) and controls (*actin-Gal4*), as determined by qPCR. To generate these flies, the ubiquitously expressed actin driver was combined with a *UAS*-*GlyP* RNAi construct (*actin-Gal4* + *UAS-GlyP-*RNAi) to knock down *GlyP* expression or crossed to the isogenic background of the RNAi construct to generate the isogenic control (*actin-Gal4*). (D) Survival curves of *GlyP* ubiquitous knockdown flies and controls (*actin-Gal4*, treated) upon paraquat-induced OS (treated) or untreated. Genotypes are as in (C). Median survival time *actin-Gal4* + *UAS*-*GlyP*-RNAi (treated): 28 h, *n* = 96 versus *actin-Gal4* (treated) 79 h, *n* = 96; *p* < 0.0001. Survival curves showing percent survival and SE over time were plotted using Graphpad, and *p*-values were obtained using the Gehan-Breslow-Wilcoxon test. The numerical data depicted in this figure can be found in S5 Data. GlyP, glycogen phosphorylase; n.s., not significant; OS, oxidative stress; qPCR, quantitative PCR; RNAi, RNA interference; SE, standard error.

First, we provided a high dose of glucose as an immediately accessible energy source (Fig 8A). This high-sugar diet restored survival of G9a mutants to control levels (G9a null versus G9a null high sugar, *p* < 0.0001; controls versus G9a null high sugar, *p* = 0.885) while also extending the survival of controls (controls versus controls high sugar, *p* > 0.0001 (Fig 8A). In contrast, when flies were provided a high-protein diet, no improvement of survival in G9a mutants or controls was observed (Fig 8B). Improved OS resistance in response to a high-sugar diet in G9a mutants and controls suggests that energy availability is generally a limiting factor in OS resistance. To directly address if limiting energy availability affects OS resistance, we determined survival of flies with ubiquitous knockdown of glycogen phosphorylase (GlyP), the rate-limiting enzyme responsible for glycogen breakdown into glucose. Ubiquitous GlyP RNAi knockdown resulted in a 66% reduction in *GlyP* mRNA (Fig 8C) and caused partial lethality. The surviving GlyP-knockdown adults recapitulated the reduced OS tolerance of G9a mutants (Fig 8D). Taken together, these data show that access to energy stores is a limiting factor in resistance to OS exposure. G9a mutants, which are characterized by a perturbed metabolic response to OS resulting in a net reduction of available energy, show reduced OS resistance.

## Discussion

In this study, we found that G9a mutants show reduced survival upon OS exposure and investigated the underlying transcriptional and physiological mechanisms. Transcriptional profiling revealed that the largest group of DE genes are highly augmented upon OS induction in the G9a mutant (41.7% of all DE genes, Group 3, Fig 2B). The second largest group of DE genes (23.9% of all DE genes, Group 5, Fig 2B) were more down-regulated in G9a mutants in response to OS. Genes that are overactivated in G9a mutants are predominantly involved in OS response and OS-mediated damage, whereas genes that are down-regulated in G9a mutants are involved in energy metabolism.

Analyses of OS markers revealed that G9a mutants were able to cope with the OS challenge, since ROS levels and OS-mediated damage were not increased in G9a mutants (Fig 4), and antioxidant supplementation did not rescue G9a mutant survival during OS (S5 Fig). Instead, G9a mutants showed abnormal energy use during OS exposure that was characterized by a rapid depletion of glycogen stores and by a failure to mobilize triglycerides (Fig 6). Death of the mutant population coincided with exhausted glycogen stores and was rescued effectively by supplying an immediately available energy source, glucose (Fig 7). Moreover, we found that energy stores also declined in control animals in response to the applied OS regime and that survival of controls also improved upon glucose supplementation, suggesting that energy availability in general is a limiting factor in OS resistance. In support of this, we demonstrated that directly limiting access to glycogen stores through knockdown of GlyP, the rate-limiting enzyme in glycogen mobilization, also causes OS sensitivity. Together, our data suggest that G9a safeguards appropriate transcriptional and metabolic responses to OS to ensure that optimal energy recourses are available to fuel the stress response.

### Mechanistic basis of OS susceptibility in G9a mutants

We propose that three mechanisms may contribute to increased OS susceptibility in G9a mutants: (1) inaccessible fat stores, (2) inefficient use and thus accelerated wasting of glycogen, and (3) an overactivated transcriptional response to OS that will consume additional energy.

At steady state, expression of the lipid phosphatases *CG11437* and *CG11438*, which are important for triglyceride synthesis, are up-regulated in G9a mutants (Fig 5K and S4B, S6 and S7 Figs). Furthermore, expression of *heimdall* and *bubblegum*, two important enzymes in charge of breaking down triglyceride long-chain fatty acids, are down-regulated in G9a mutants (Fig 5H and S4B, S6 and S7 Figs). These findings may account for the identified high triglyceride stores and increased abundance and size of fat body lipid droplets in G9a mutants at steady state (Fig 6B and 6C). *Heimdall* and *bubblegum* expression levels remain low in G9a mutants during OS exposure, whereas in controls *heimdall* is strongly induced, which could explain the defect in lipid mobilization during the OS response. Three transcription factors are known to bind to both *heimdall* and *bubblegum* cis-regulatory elements (dorsal, kruppel, and medea [31]), but none of them has been linked to G9a, and in general little is known about G9a’s sequence-specific cofactors.

Whereas triglycerides remain inaccessible in G9a mutants, we observed a rapid exhaustion of glycogen stores, suggesting that there is a high energy demand during the OS response. Depletion of glycogen stores during OS exposure has been reported previously [32]. Energy is needed for the rapid production and activity of ROS protective enzymes. In the absence of G9a, ROS protective enzymes are overactivated (Fig 3). Our data argue that this inappropriate scaling of the transcriptional response to OS causes an additional energy demand in G9a mutants that is exacerbated by their deficiency in triglyceride breakdown, resulting in early death due to lack of available energy. Some of the overactivated genes, such as *GstE1*, *Prx2450-1*, *Cat*, and *Ldh*, were previously identified as targets for G9a-mediated H3K9me2. This suggests that this epigenetic modification might serve to buffer stress-induced gene activation, as we have previously observed in the response to viral infection [16]. However, these epigenetic differences were identified originally in whole larvae, and it remains to be seen if chromatin changes are also present in G9a mutant heads or in other specific tissues/cells that we identified to be important, such as the fat body or dilp2-expressing neurons.

Our data further indicate that G9a mutants may suffer from an OS-induced metabolic shift resulting in energy wasting, known as the Warburg effect or aerobic glycolysis [33]. The rapid use of glycogen (Fig 6A) and the highly increased expression of *Ldh* in *G9a* mutants under stress conditions (Fig 5M) are consistent with this metabolic state that is often seen in tumors, in which all energy is derived from glycolysis rather than from mitochondrial oxidative phosphorylation, despite the presence of oxygen. Under these conditions the main product of glycolysis, pyruvate, is converted to lactate by Ldh, a process that produces energy quickly but less efficiently than mitochondrial oxidative phosphorylation.

### G9a functions as protective factor in various stress responses

Recently, it was suggested that G9a is required for optimal survival in response to starvation in *Drosophila* [17]. An and colleagues [17] proposed that the role of G9a is highly specific for this type of stress, as they were unable to detect susceptibility of G9a mutants to heat stress and observed no difference in survival time upon exposure to 10 mM paraquat when compared to controls. In contrast, we demonstrate the importance of G9a in the transcriptional and physiological response to OS and have previously provided evidence for G9a’s role in the response to virus infections [16]. In addition, we have observed that G9a-deficient flies are sensitive to several stresses in addition to paraquat, including the alternative potent OS-inducing agent MSB (S1B Fig) and other stressors such as heat stress (S10A Fig) and cold shock (S10B Fig). Our observations are supported by several recent cell culture studies indicating the importance of G9a in hypoxia resistance during the rapid proliferation of cancer cell lines [14,15]. Thus, in contrast to previous conclusions [17], it appears that *Drosophila* G9a has a protective function in response to multiple different types of stress. Another recent study has shown that G9a mutants show increased resistance to starvation on agar media [34], which is consistent with our own findings (S10C Fig) and makes sense in light of the increased steady-state energy stores. The inconsistencies with An and colleagues [17] might arise from methodological differences that were employed by the authors, such as the use of high temperature (29°C) in combination with using filter paper during starvation assays, as opposed to agar media. It is conceivable that these conditions might result in confounding factors such as dehydration.

Here, we report reduced survival of two G9a-null mutants and G9a RNAi-mediated knockdown flies upon exposure to 50 mM paraquat, which induces lethality within 48 h (Fig 1). An and colleagues concluded that G9a is not important for OS resistance. Their applied level of OS using 10 mM paraquat appears to be mild, as flies survived for up to 20 d [17]. This may suggest that G9a is particularly essential to defeat high levels of OS, which may be supported by our observation that G9a was not required for OS resistance when exposed to 5% H_2_O_2_ (S10D Fig), a milder OS agent than paraquat. It is also compatible with our finding that G9a mutants are well able to combat ROS and oxidative damage, even under high OS as tested in our study, and that decreased OS resistance results from energy shortage. An and colleagues also observe that G9a mutants have a higher level of glycogen in steady-state conditions, which is rapidly depleted in response to starvation. They did not observe increased triglycerides in G9a mutant flies, nor did they observe an inability to access these stores in response to starvation, findings for which we provided evidence both in histology as well as in metabolite assays (Fig 6). Although the discrepancy at steady state may relate to the different G9a mutant that they have utilized, triglyceride usage in G9a mutants may also be specific to the type of stress. Taken together, these studies suggest that G9a is involved in a complex metabolic response to multiple types of environmental stress.

### Potential relevance of the identified transcriptional and metabolic dysregulation to human disease

Recent studies have shown that G9a dampens expression of target genes regulating hypoxia, tumor suppression, autophagy, and angiogenesis, making it an attractive target to interfere with cancer progression [14,15]. Of note, mutations in the *G9a* orthologue *EHMT1* in humans cause Kleefstra syndrome, a neurodevelopmental disorder that is characterized by intellectual disability and autism [24,35] and that also shows neurodegenerative features [36]. Whether the disorder is characterized by increased cancer resistance and/or metabolic defects is unknown, but increased frequency of obesity has been reported as a feature of Kleefstra syndrome [37,38]. Combined loss of *Drosophila heimdall* and *bubblegum*, the two key enzymes of triglyceride long-chain fatty acid breakdown that we found to be highly down-regulated in G9a mutants, causes neurodegeneration [39], raising the possibility that equivalent metabolic mechanisms could underlie neurodegenerative features of Kleefstra syndrome [36]. Our data propose metabolic dysregulation as a novel hypothesis about the mechanisms underlying physiological and pathological aspects of Kleefstra syndrome, meriting novel lines of clinical investigation.

In conclusion, we have identified a role for *Drosophila* G9a in the transcriptional and metabolic response to systemic OS exposure. These findings were obtained by feeding adult *Drosophila* a lethal dose of paraquat. Paraquat has been used as a herbicide in agriculture and adverse effects on health due to chronic exposure are well studied in humans [40]. Although our experimental conditions do not mimic the environmental exposure in human populations, it has allowed us to identify the mechanistic role of G9a as a generally protective factor in the organismal response to environmental stress.

## Materials and methods

### Fly stocks and maintenance

Flies were reared on standard medium (cornmeal/sugar/yeast) at 25°C and 70% humidity on a 12 h light/dark cycle. Flies were reared at 28°C and 60% humidity for tissue-specific G9a RNAi-mediated knockdown. *G9a*^*DD1*^ and *G9a*^*DD2*^ mutants were generated previously by P-element excision [22]. Although both mutants show undetectable G9a protein by western blot, *G9a*^*DD2*^ shows slightly milder phenotypes than *G9a*^*DD1*^ [22], indicating that *G9a*^*DD2*^ may be a strong hypomorph rather than a G9a-null allele. A precise transposon excision line has been generated in the same genetic background and served as a control for *G9a*-null mutants in all experiments. After verification of OS survival in *G9a*^*DD1*^ and *G9a*^*DD2*^ (Fig 1A and S1A Fig), we continued further experiments using *G9a*^*DD1*^, referred to as G9a-null mutant throughout the paper. The following driver lines were obtained from the Bloomington *Drosophila* stock center (Indiana University) *actin-Gal4*, *lsp2-Gal4*, *dilp2-Gal4*: *yw; actin-Gal4/CyO* (BL4414), *yw;; lsp2-Gal4* (BL6357), *w*^*1118*^*; dilp2-Gal4/CyO* (BL37516). The driver *w*^*1118*^*; UAS-Dicer-2/CyO GFP; elav-Gal4/TM6C* was assembled in house. To generate the G9a- and GlyP-knockdown progenies and their isogenic controls, the drivers were crossed to the *UAS-G9a-RNAi* (*w*^*1118*^*; UAS-G9a-RNAi/CyO* [VDRC25474]) and *UAS-GlyP-RNAi* (*w*^*1118*^*; UAS-GlyP-RNAi/CyO* [VDRC27928]) lines and to the isogenic background of the two RNAi lines (*w*^*1118*^ [VDRC60000]). VDRC25474, VDRC27928, and VDRC60000 lines were obtained from the Vienna Drosophila Resource Center (VDRC).

### Paraquat exposure and sample collection

Flies were collected after eclosion and allowed to recover from CO_2_ exposure for 5 d prior to paraquat exposure. Paraquat (Methyl viologen dichloride hydrate 98%; Sigma 856177) was mixed into the fly food at 40°C to a final concentration of 50 mM. For OS induction, 5–9 d old flies were transferred to paraquat-containing food and incubated at 25°C and 70% humidity. At each time point, flies were flash frozen in liquid nitrogen followed by vortexing and filtering through a series of sieves to isolate heads from other body parts. Fly heads were used for RNA extraction and metabolic measurements.

### Paraquat survival assay

TriKinetics *Drosophila* Activity Monitors (DAM2) were used to quantify survival during paraquat exposure. Flies (5–9 d old) were allowed to recover from CO_2_ exposure for 5 d. They were transferred by aspiration into 5 mm diameter tubes containing normal food or food supplemented with 50 mM paraquat and incubated at 25°C and 70% humidity. Raw activity monitor files were processed using DAMFileScan110 software (TriKinetics). Monitor counts were binned per hour, and time of death was determined when counts reached zero. Survival curves showing mean survival over time were plotted using Graphpad Prism. *p*-Values were obtained using the Gehan-Breslow-Wilcoxon test. Experiments were repeated at least three times.

### Quantification of food intake

Food intake was quantified using the automated, high-resolution behavioral-monitoring system flyPAD [41]. Fully fed individual male flies were placed in flyPAD arenas for 1 h with standard food containing 1% agarose and 50 mM paraquat, following 0, 6, or 12 h of paraquat treatment. Total number of sips, previously shown to be the most reliable measure of food intake [41], was used.

### Treatment and analysis of responses to other stressors

For heat stress, two groups of 20 flies in standard food vials were put in a water bath at 37°C. For cold shock, five groups of 20 flies in empty vials were put in a salt brine ice bath at −5°C for 1 h and moved to standard food vials for recovery. For MSB (Sigma M5750), flies were exposed to 75 mM MSB-containing food. For starvation, 16 individual flies per genotype were transferred into vials containing 1% agar. For H_2_O_2_, five groups of 20 flies were put on fly food containing 5% H_2_O_2_. Survival was monitored manually or using DAM activity monitors (Materials and methods subsection Paraquat survival assay). During all stress assays except heat stress, survival was monitored at 25°C and 70% humidity. All experiments were repeated at least twice. Survival curves showing percent survival over time were plotted using Graphpad, and *p*-values were obtained using the Gehan-Breslow-Wilcoxon test.

### High-sugar and high-protein diet

Food was prepared with four-times-increased sugar dose (440 g/liter) or two-times-increased dry baker’s yeast (56 g/liter) to obtain a high-sugar or high-protein diet, respectively. Flies were transferred to vials containing either of these diets and 50 mM paraquat and monitored for survival. Survival curves showing percent survival over time and SE were plotted using Graphpad, and *p*-values are obtained using the Gehan-Breslow-Wilcoxon statistical test. Experiments were repeated three times.

### Antioxidant treatment

Standard food was prepared, and paraquat was added to a final concentration of 50 mM. Then, 0.5 mM vitamin E (α-Tocopherol, Sigma 258024) and 0.25 mM reduced glutathione (L-Glutathione, Sigma G4251) were added to paraquat-containing food, and flies were subsequently monitored for survival. Survival curves showing percent survival over time were plotted using Graphpad, and *p*-values are obtained using the Gehan-Breslow-Wilcoxon statistical test. Experiments were repeated two times.

### RNA-seq and data analysis

RNA was extracted from 200 fly heads per sample using QIAGEN lipid mini tissue kit. The TruSeq RNA Sample Preparation Kit (Illumina) was used to prepare adapter ligated PCR fragments for sequencing. In brief, mRNA was purified from total RNA and fragmented. The cleaved mRNA was primed with random hexamers and reverse transcribed into first-strand cDNA. The RNA template was then removed, and a replacement, complementary strand was generated. The ends of the double-stranded cDNA were repaired and adenylated. Then, sequencing adapters were ligated to the prepared cDNA. PCR was used to selectively enrich the fragments containing the adapters. The PCR fragments were validated using Agilent 2200 TapeStation. Single indexed samples were multiplexed and sequenced on an Illumina HiSeq 2500 sequencing system in single-end mode with a read length of 30 bp. Quality of sequenced reads was assessed with FastQC. The RNA-seq experiments were conducted on two biological duplicates for each condition. Sequenced reads were aligned with the Burrows-Wheeler algorithm (BWA) [42] to the *Drosophila* reference genome (BDGP 5), and per-gene read counts were generated with HTSeq count [43]. A total of 25–30 million reads with high-quality alignment were obtained for each sample and used for differential expression analysis (S1 Data). DESeq [25] was used to obtain library size–normalized read counts and to generate heatmap and principle component plots. DE genes (fold change ≥ 1.5, adjusted *p*-value ≤ 0.05, Benjamini-Hochberg) were identified using DESeq in seven pairwise comparisons: control 0 h versus 6 h, control 0 h versus 12 h, G9a mutant 0 h versus 6 h, G9a mutant 0 h versus 12 h, G9a mutant versus control 0 h, G9a mutant versus control 6 h, and G9a mutant versus control 12 h after OS exposure (Fig 2, S3 Fig and S2 Data). The RNA-seq data are available at the NCBI Gene Expression Omnibus under series accession number GSE110240.

### RT-qPCR

RNA was isolated from fly heads in triplicate using the RNeasy Lipid Tissue Mini Kit (QIAgen), with DNase treatment, and cDNA synthesis was performed using iScript Reverse Transcription Supermix or the SensiFAST cDNA Synthesis Kit (Bioline). RT-qPCR was performed using a 7500 Fast Real-Time PCR System (Applied Biosystems) and the BioRad CFX 384 with the GoTaq Green Master Mix (Promega) or SensiFAST SYBR No-Rox kit. Expression of target genes was normalized to transcript levels of the reference genes *betacop*, *gamma-tubulin 23C*, and *eIF2*. Detections of GlyP RNAi-mediated knockdown was done as previously described [44]. All primers (S4 Data) were validated for efficiency according to standard procedures.

### Clustering and GO

Clustering was performed using the PAM algorithm [26]. DE genes were clustered based on log2 fold changes in the four different pairwise comparisons. GO analysis was performed on the five principle groups (Fig 2C) using Panther (http://pantherdb.org/) [45] with all *Drosophila* genes as the background and with Bonferroni-corrected *p*-values. Annotation of enzymes involved in ROS (Fig 3) and metabolic pathways (Fig 5) is based on GO as well as manual annotation of known enzymes involved in these processes. A complete list of genes and gene expression values for the different ROS and metabolic pathway groups shown in Figs 3 and 5 is provided as S3 Data.

### Metabolite analysis

Groups of 20 fly heads in triplicate were used for metabolic measurements. H_2_O_2_, lipid peroxidation, and triglyceride and glycogen levels were measured using H_2_O_2_ colorimetric assay (K265), lipid peroxidation (MDA) colorimetric assay (739), triglyceride quantification colorimetric assay (K622), and glycogen colorimetric assay kit II (K648), respectively, according to the manufacturer’s protocols (BioVision). Protein level was measured in parallel for each sample using Pierce BCA Protein Assay Kit, and metabolite levels were normalized to total protein content. Bar graphs showing mean values and standard error of the means (SEMs) were generated using Graphpad, and *p*-values were obtained using multiple *t* tests followed by FDR correction according to Benjamini, Krieger, and Yekutieli.

### Microscopy sample preparation and imaging

Fly heads were collected in 2% glutaraldehyde buffered with 0.1 M sodium cacodylate (pH 7.4), postfixed in 1% osmium tetroxide in Palade buffer (pH 7.4) with 0.5% potassium hexacyanoferrate(III)-trihydrate and after dehydration in ethanol and propylene oxide. Drops of 5% triton X-100 in PBS were added to decrease surface tension. Heads were embedded in EPON epoxy resin and fixed at 50°C overnight. Embedded fly heads were cut transversally with a microtome blade to the plane where optical lobes connect to the central brain, for consistency between sections. For light-microscopy imaging, semithin slices (1 μm) were cut and subsequently stained with toluidine blue, and images were captured at 10× magnification using an Axioskop 2 plus microscope. For scanning electron microscopy images, ultrathin sections (±80 nm) are made and contrasted with 6% uranyl acetate and lead citrate solutions. Images were captured on a JEOL 6310 SEM.

## Supporting information

S1 Fig G9a mutants show reduced survival to OS. (A) Survival curves of *G9a*^*DD2*^ mutants and controls upon paraquat-induced OS exposure (treated) show reduced survival in *G9a*^*DD2*^ mutants (median survival time: *G9a*^*DD2*^ mutants, treated: 48 h, *n* = 57; versus control, treated, 72 h, *n* = 57; *p* < 0.0001). G9a mutants and controls show normal longevity without OS exposure (untreated) during the time course of the experiment (*G9a*^*DD2*^, untreated *n* = 118; control, untreated: *n* = 118). Survival curves showing percent survival over time and SE were plotted using Graphpad, and *p*-values are obtained using the Gehan-Breslow-Wilcoxon statistical test. (B) Survival curves of G9a-null mutants and controls upon MSB-induced OS exposure show reduced survival in G9a mutants (median survival time: *G9a*^*DD1*^ mutants, treated: 33 h, *n* = 80; versus control, treated, 58 h, *n* = 80; *p* < 0.0001). Survival curves showing percent survival over time and SE were plotted using Graphpad, and *p*-values are obtained using the Gehan-Breslow-Wilcoxon statistical test. The numerical data depicted in this figure can be found in S5 Data. MSB, menadione sodium bisulfide; OS, oxidative stress; SE, standard error.
(EPS)

S2 Fig Comparison of mRNA expression profiles (RNA-seq) from G9a mutants and controls during OS exposure. (A) Heatmap shows Euclidean sample-to-sample distances of RNA-seq OS samples, using normalized count data. Phylogenic tree on top of the figure shows Euclidean distance of OS samples; corresponding sample names are shown at the bottom. The color key indicates correlation coefficient of one sample (y-axis) to the other sample (x-axis). DESeq-normalized reads per gene obtained from RNA-seq were compared between samples. (B) Principal component analysis showing the clustering of biological replicates in the two largest components contributing to sample variance. OS sample conditions are shown on top of the figure, and corresponding dots represent variance of individual samples compared to all other samples. Note that the two control sample data points (in red) are overlapping. The numerical data depicted in this figure can be found in S5 Data. OS, oxidative stress; RNA-seq, RNA sequencing.
(EPS)

S3 Fig G9a mutants show highly augmented transcriptional response of genes regulating stress defenses and metabolism (alternative comparison). This figure shows an alternative analysis approach to Fig 2. Here, expression values of the *G9a*^*DD1*^ mutants are compared to the controls at the different time points. (A) Partitioning around medoids clustering of differentially expressed genes based on log2 fold change values obtained from differential expression analysis in alternative three pairwise comparisons. (B) Heatmap and boxplots of log2 fold changes of differentially expressed genes combined into five principle groups derived from clusters with similar patterns of differential expression. The five principle groups show up-regulation in G9a mutants under all OS conditions (group 1, clusters 1–3), down-regulation in G9a mutants under all OS conditions (group 2, clusters 4–6), up-regulation in G9a mutants only after 6 and 12 h OS (group 3, clusters 7 and 8), up-regulated in G9a mutants at 0 h OS and down regulated in G9a mutants after OS exposure (group 4, clusters 9 and 10), and more down-regulation in G9a mutants after OS exposure (group 5, clusters 11 and 12). The number of genes in each group is indicated. (C) Gene ontology analysis showing the top enriched biological processes sorted by adjusted (Bonferroni-corrected) *p*-value in each of the five principal groups, indicating enrichment in stress response genes (highlighted in yellow) and metabolic genes (highlighted in brown). The numerical data depicted in this figure can be found in S5 Data.
(EPS)

S4 Fig Validation of ROS and metabolic RNA sequencing expression values by qRT-PCR. (A) Bar graph of differentially expressed genes in G9a mutants and control involved in ROS or metabolic pathways during 0, 6, and 12 h OS exposure. Bars show log2 fold change values with SEM, and *p*-values are obtained using two-way ANOVA. (B) qPCR validation of a set of metabolic genes; bars show log2 fold change values with SEM, and *p*-values are obtained using Student *t* test. *p = 0.01; **p = 0.001; ***p = 0.0001; ****p < 0.0001; n.s., *p* > 0.05. (C) Scatterplot showing a strong correlation between log2 fold change values obtained from RNA sequencing (y-axis) and qRT-PCR (x-axis). Linear regression value is 0.85, and Pearson correlation value is 0.92. The numerical data depicted in this figure can be found in S5 Data. n.s., not significant; OS, oxidative stress; qRT-PCR, quantitative real-time PCR; ROS, reactive oxygen species.
(EPS)

S5 Fig No improvement of the OS-induced *G9a* survival deficiency by antioxidant treatments. (A, B) Survival curves show of G9a mutants and controls fed with vitamin E (0.5 mM) (A) or GSH (0.25 mM) (B) during OS exposure. (A) Vitamin E–treated G9a mutants (G9a null Vitamin E) and controls (control Vitamin E) show no improvement compared to only paraquat-treated flies. (B) GSH-treated G9a mutants (G9a null GSH) and controls (Control GSH) show no improvement compared to only paraquat-treated flies. Survival curves showing percent survival over time and SE were plotted using Graphpad, and *p*-values are obtained using the Gehan-Breslow-Wilcoxon test. The numerical data depicted in this figure can be found in S5 Data. GSH, glutathione; OS, oxidative stress; SE, standard error.
(EPS)

S6 Fig G9a mutants show dysregulation of triglyceride synthesis and lipid breakdown genes at steady state and during OS exposure. Boxplots showing log2 fold changes for selected groups of genes encoding enzymes involved in (A) triglyceride synthesis and (B) fatty acid beta oxidation. Genes *CG11437* and *CG11438* (red circles) as well as *bubblegum* (blue) and *heimdall* (green) are highlighted as highly dysregulated genes (circles and arrows). The numerical data depicted in this figure can be found in S5 Data. OS, oxidative stress.
(EPS)

S7 Fig Gene expression changes in metabolic pathways in G9a mutants during OS exposure. (A-K) Boxplots showing log2 fold changes for selected groups of genes encoding enzymes involved in glycogen breakdown (A), glycogen synthesis (B), gluconeogenesis (C), glycolysis (D), pyruvate dehydrogenases (E), citric acid cycle (F), fatty acid beta oxidation (G), ketogenesis (H), ketolysis (I), triglyceride synthesis (J), and mitochondrial oxidative phosphorylation (K). Fold changes were derived from the following pairwise comparisons: G9a mutant versus control at 0 (beige), 6 (orange), and 12 h (dark orange) of OS. The numerical data depicted in this figure can be found in S5 Data. OS, oxidative stress.
(EPS)

S8 Fig G9a mutants have normal food intake both at steady state and during OS exposure. Food intake is indicated as the cumulative number of total sips, as measured using the FlyPAD assay, at 0, 6, and 12 h of OS. *p*-Values are obtained using 2-way ANOVA; n.s., *p* > 0.05. The numerical data depicted in this figure can be found in S5 Data. n.s., not significant; OS, oxidative stress.
(EPS)

S9 Fig Panneuronal G9a knockdown does not significantly affect survival during OS. Survival curves of panneuronal G9a knockdown (*UAS-dicer2*; *elav*-Gal4 + *UAS-G9a-*RNAi) during OS exposure does not result in a significant reduction of survival compared to the controls (*UAS-dicer2*; *elav-Gal4*) (median survival time: *UAS-dicer2; elav-Gal4* + UAS-*G9a*-RNAi, 24 h, *n* = 32 versus *UAS-dicer2; elav-Gal4*, 23.5 h, *n* = 32; *p* = 0.071). The panneuronally expressed elav driver was combined with a *UAS-G9a* RNAi construct (*UAS-dicer2*; *elav-Gal4* + *UAS-G9a-*RNAi) to knock down G9a expression or crossed to the isogenic background of the RNAi construct to generate the isogenic control (*UAS-dicer2; elav-Gal4*). Survival curves showing percent survival over time and SE were plotted using Graphpad, and *p*-values are obtained using the Gehan-Breslow-Wilcoxon test. The numerical data depicted in this figure can be found in S5 Data. OS, oxidative stress; RNAi, RNA interference; SE, standard error.
(EPS)

S10 Fig Response of G9a mutants to other stressors. (A-B) Survival curves show reduced survival of G9a mutants during heat stress (37°C, *p* < 0.001, *n* = 55) (A) and cold shock recovery (−4°C, *p* < 0.001, *n* = 130) (B), compared to controls. (C) G9a mutants are less sensitive to starvation and survive longer than controls (*p* = 0.0023, *n* = 16). (D) Survival of G9a mutants on hydrogen peroxide is not significantly decreased compare to controls (*p* = 0.0621, *n* = 225). G9a mutants in (A): *G9a*^*DD2*^, all other panels: *G9a*^*DD1*^. Survival curves showing percent survival over time and SE were plotted using Graphpad, and *p*-values are obtained using the Gehan-Breslow-Wilcoxon test. The numerical data depicted in this figure can be found in S5 Data. SE, standard error.
(EPS)

S1 Data Read count and alignment statistics for RNA sequencing. (XLSX)

S2 Data Differential expression and clustering statistics for RNA-seq data in Fig 2 and S3 Fig. RNA-seq, RNA sequencing.
(XLSX)

S3 Data Gene ontology enrichment statistics for the five principle groups in Fig 2 and S3 Fig. (XLSX)

S4 Data Primer sequences of genes used for RT-qPCR. RT-qPCR, quantitative real-time PCR.
(XLSX)

S5 Data Numerical values of underlying figures. (XLSX)

## Abbreviations

Atf-3: activating transcription factor 3
AP-1: activator protein 1
*Cat*: *catalase*
ChIP-seq: chromatin immunoprecipitation sequencing
DE: differentially expressed
dilp: *Drosophila* insulin-like peptide
EHMT: euchromatin histone methyltransferase
FoxO: forkhead box O
GlyP: glycogen phosphorylase
GO: gene ontology
*GstE1*: *glutathione S-transferase E1*
H_2_O_2_: hydrogen peroxide
H3K9me2: histone H3 lysine 9 dimethylation
JNK: c-Jun N-terminal kinase
*Ldh*: *lactate dehydrogenase*
MDA: malondialdehyde
MSB: menadione sodium bisulfide
ngu: normalized glycogen units
ntu: normalized triglyceride units
OS: oxidative stress
PAM: partitioning around medoids
*Prx2450-1*: *peroxiredoxin 2540–1*
RNAi: RNA interference
RNA-seq: RNA sequencing
ROS: reactive oxygen species
RT-qPCR: quantitative real-time PCR
SOD: superoxide dismutase
